# The Relationship Between Feedback Quality, Perceived Organizational Support, and Sense of Belongingness Among Conscientious Teleworkers

**DOI:** 10.3389/fpsyg.2022.806443

**Published:** 2022-04-06

**Authors:** Yanyan Liu, Nan Xu, Qinghong Yuan, Zhaoyan Liu, Zehui Tian

**Affiliations:** ^1^Business School, Nankai University, Tianjin, China; ^2^School of Management, Guangzhou City University of Technology, Guangzhou, China

**Keywords:** feedback quality, sense of belongingness, perceived organizational support, conscientiousness, teleworker

## Abstract

The belongingness literature has largely examined the antecedents of non-teleworkers’ sense of belongingness, but little attention has been paid to what job-related factors could affect teleworkers’ sense of belongingness. Grounded in organizational support theory, our research focuses on why feedback quality from the direct leader brings sense of belongingness and considers how conscientiousness of teleworkers shapes this effect. Based on data from 329 participants obtained at three different time points from one technology service organization in China, our results indicated that teleworkers’ perceived organizational support serves as an essential mediator of the positive relationship between feedback quality from the direct leader and sense of belongingness. Additionally, the teleworkers’ conscientiousness strengthened the positive direct effect of feedback quality on perceived organizational support and the indirect effect on sense of belongingness. The moderating role of conscientiousness in strengthening the link between feedback quality and perceived organizational support was significant for high levels of conscientiousness and not significant for low levels. Finally, we discussed theoretical and practical implications.

## Introduction

Teleworkers refer to individuals working from locations away from their primary offices, such as home, client sites, or shared office space (Raghuram et al., 2019; Adamovic et al., 2021). Teleworking is a widely popular work mode that has been experiencing rapid worldwide growth due to its potential benefits, such as better work-life balance, reduced travel time, schedule flexibility, autonomy, and job satisfaction (Allen et al., 2015; Field and Chan, 2018; Wang et al., 2020). Although it can bring many benefits to teleworkers, the loss of organizational trappings, and spontaneous, face-to-face interaction with other organizational members makes it harder for teleworkers to maintain a salient connection with the organization (Ashforth, 2020). Thus, teleworkers are more likely to experience a lack of sense of belongingness (Belle et al., 2015; Mogilner et al., 2018), which refers to “the experience of personal involvement in a system or environment so that persons feel themselves to be an integral part of that system or environment” (Hagerty et al., 1992, p. 173) and serves as a crucial influencer of employee satisfaction and work outcomes (Randel et al., 2018; Zheng et al., 2020).

Scholars have a long history of research on sense of belongingness and found that many factors may inhibit or enhance employees’ sense of belongingness, such as physical and social isolation (Bartel et al., 2012; Kossek et al., 2015; Wang et al., 2020), leadership (Cai et al., 2018; Yang et al., 2020), and organizational support (Haines et al., 2002; Chen et al., 2020). Nevertheless, previous research mainly focuses on non-teleworkers at the workplace. Little attention has been paid to how work-related factors influence teleworkers’ sense of belongingness. Therefore, this paper will take this as the research object. Among studies that addressed the antecedents of sense of belongingness, organizational support is considered as a key motivator for individuals to identify their intention to belong (Casimir et al., 2014; Chen et al., 2020). Specifically, due to the constraints of the work environment, teleworkers’ interactions with the organization (i.e., tackling the possible problems and challenges arising in teleworking) are primarily from dyadic interactions with their leaders at work (Park and Cho, 2020). In the daily two-way communication between leaders and employees, leaders providing job feedback to employees are an important part of the process (Ashford et al., 2016). Therefore, feedback becomes a particularly important source for teleworkers to feel the support of organizations (Kumar et al., 2018; Guan and Frenkel, 2020). However, despite the importance of feedback to teleworkers, few studies have focused on the relationship between feedback from leaders and teleworkers’ sense of belongingness. Feedback quality, as one of the most practical aspects of feedback, refers to relevant, specific, and detailed information to make their job performance progress (Steelman et al., 2004), and determines the extent to which feedback can help work progress. Thus, this study will focus on how and when feedback quality affects teleworkers’ sense of belongingness.

Specifically, organizational support theory suggests that helping employees in stressful situations or helping employees deal with their jobs effectively are believed to be the assurance of organizational support for employees, and such support will catalyze positive employee outcomes (Ahmed et al., 2015; Islam et al., 2019). We believe that delivered feedback from leaders can be regarded as a way for organizations to support their teleworkers, which in turn can evoke their sense of belongingness. As demonstrated in previous studies, supervisors act as agents of the organization (Eisenberger et al., 2002; Jin and McDonald, 2017), thus employees in generally view their supervisor’s favorable treatment (i.e., high-feedback quality) toward them as indicative of the organizational support (Baran et al., 2012; Kurtessis et al., 2017). Based on this reason, we believe that organizational support may be the intrinsic mechanism connecting feedback quality and teleworkers’ sense of belongingness.

To further identify the boundary conditions of how feedback quality affects sense of belongingness *via* perceived organizational support, personality traits are considered as important factors influencing individuals’ reactions to and use of feedback (Furnham, 1989; Smither et al., 2005). In this study, teleworkers are not under the daily visible supervision of their leaders, so we chose conscientiousness to explore when these relationships change. Conscientious individuals are described as hard-working, persistent, achievement-striving, and goal-oriented (Barrick et al., 2005; Singh, 2019; Tu et al., 2020). Individuals with high conscientiousness may work harder to address and deal with feedback than those with low conscientiousness because they have a greater willingness to achieve and are more focused or self-disciplined in completing tasks (Cianci et al., 2010). Thus, we believe that highly conscientious individuals place more emphasis on feedback to meet the challenges of being away from the organization. With this, they are more likely to view high-quality feedback as support from the organization, which in turn reinforces the individual’s sense of belongingness. Therefore, we expect that conscientiousness moderates the relationship between feedback quality and sense of belongingness through perceived organizational support.

**Figure 1:**
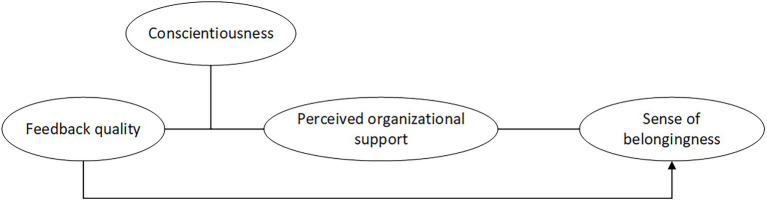
The research model.

This research makes some critical theoretical contributions. First, the present study enriches the literature on sense of belongingness by targeting teleworkers, who form a prevalent workgroup in current society have rarely been studied, and by identifying feedback quality as an antecedent. Second, drawing from organizational support theory, we reveal the mediating role of perceived organizational support between feedback quality and sense of belongingness. Finally, this study will further delineate the boundary conditions of the hypothesized relationship by examining the moderation of conscientiousness. We propose that the level of conscientiousness may directly affect their attitude toward feedback. Such investigation provides a more nuanced picture of when feedback quality influences teleworkers’ sense of belongingness, allowing us to better understand who is more sensitive to feedback quality for teleworkers. Figure 1 depicts our overall research model.

## Theory and Hypotheses Development

### Feedback Quality and Sense of Belongingness

Belongingness is a mental health concept that describes the self as a perceived part of an organization (Hagerty et al., 1992) and is often seen as the result of frequent, pleasurable interactions with others (i.e., follower–leader; Baumeister and Leary, 1995; Chan et al., 2015; Kia et al., 2019). Teleworkers are more likely to suffer from a lack of belongingness because they have to meet the challenge of remaining connected to the organization while working outside of the organization. As a result, they are more likely to feel that there is no community to rely on for support, so that they are more likely to feel isolated and invisible, as well as to no longer maintain a sense of belongingness with the organization and eventually lose their intimate connection to the organization (Belle et al., 2015). However, in the frequent daily communication between teleworkers and the organization, in addition to the organization’s task, assignment to teleworkers is the organization’s daily work feedback to teleworkers. Therefore, the quality of feedback may affect the employee’s perception of the relationship with the organization and satisfaction with work interactions.

Specifically, as Xanthopoulou et al. (2012) argued, daily job resources (i.e., high-quality feedback) could predict employees’ positive emotions because those resources concern individuals’ sense of their ability to successfully control their environment. In this study, for teleworkers, helpful feedback from the supervisor is a kind of important work resources, which refers to job aspects that are functional in achieving work goals and stimulate personal growth and development (Bakker and Demerouti, 2007). Thus, we believe that high-quality feedback can induce positive emotions in the internal interactions of the organization. Low-quality feedback can leave teleworkers believing their leaders are not helping them effectively, which can breed anxiety and helplessness (Reimann and Guzy, 2017). Feelings of helplessness, especially for teleworkers, pose a threat to his status and safety within the organization and adversely affect individuals’ sense of belongingness at work (Hershcovis et al., 2017), and then withdraw from the organization (Chan et al., 2015; O’Reilly et al., 2015; Randel et al., 2018). A similar study found that when employees have not received the feedback they need, it will hurt their need to belong or connect to others (McIlroy et al., 2021). Thus, we propose that high-feedback quality can increase teleworkers’ sense of belongingness. Instead, if employees perceive their organization as not treating them positively (e.g., low feedback quality), they are less likely to become attached to the organization and stay with it (Joo et al., 2015). Thus, we believe as:

*Hypothesis 1:* Feedback quality will be positively associated with teleworkers’ sense of belongingness.

### The Mediating Role of Perceived Organizational Support

According to perceived organizational support theory, perceived organizational support refers to employees’ general beliefs about the degree to which their organization values their contributions and cares about their wellbeing (Shanock and Eisenberger, 2006, p. 206). Organizational literature also indicates that organizational support signifies that employees are cared for and valued (Tremblay and Gibson, 2016). Since leaders are viewed as agents of the organization, employees tend to perceive the leaders’ behaviors as how much the organization values their contributions and wellbeing (Eisenberger et al., 1986). Direct leaders typically have more frequent daily contact with employees, so they have more opportunities to demonstrate support (or lack thereof) to employees than organizations do (Wu and Parker, 2017).

Previous research suggests that feedback usually is regarded as an indicator of organizational support (McIlroy et al., 2021). Feedback quality holds a valuable resource to help employees attain work tasks and fulfill job responsibilities associated with their specific position at work. In reality, employees wait not passively for feedback but actively seek it in casual daily interactions at work (Ashford et al., 2016; Lam et al., 2017; De Stobbeleir et al., 2020), which expresses the extent to the desire of employees for feedback. For teleworkers who work remotely, high-quality feedback is typically characterized by the perceived consistency and usefulness of the feedback, and therefore involves the informational value of the feedback message (Dahling et al., 2015; Wang et al., 2015). By contrast, low-quality feedback may be less likely to make teleworkers feel cared for and valued by their leaders because of its inefficiency in helping them improve their work. Thus, making teleworkers perceive organizational support by providing them with high-feedback quality will result in their stronger identification and commitment to the organization, which will ignite teleworkers’ enthusiasm to help the organization succeed and help them achieve a greater sense of psychological wellbeing (Kurtessis et al., 2017). This will act as a signal to employees that they can count on the organization to help them when job demands are great (McIlroy et al., 2021). Some empirical studies have shown that managers engaging in encouraging others, nurturing others, and endeavoring to assist others’ development will motivate employees’ perception of organizational support (Zhou and Miao, 2014).

Furthermore, several recent studies have examined how perceived organizational support contributes to employees’ sense of belongingness. Park et al. (2016) found that providing support, direction, and feedback regarding career plans and personal development to the employee by mentors will promote their perceived organizational support and further reduce their intention to leave. Toker et al. (2015) suggest that the instrumental and emotional support employees receive at work provides sense of belongingness due to its value of helping employees to better cope with the dual pressures of work demands. Hoppe et al. (2017) also argued that offering instrumental and informational support to employees may enhance their sense of belongingness, as those support is valued by employees and contributes to expansion of employees’ resource reservoir. The leader is a representative of the authority in an organization (Marstand et al., 2017), and authority means adequate resources. Leader support can bolster employees’ sense of belongingness because of the important inducement of resources adequacy in promoting sense of belongingness (Nifadkar and Bauer, 2016).

Altogether, the type of treatment an employee receives from the organization is perceived to be illustrative of the employees’ position within the organization (Zagenczyk et al., 2013). Positive treatments by the organization (i.e., high-feedback quality) may symbolize an appreciated position of teleworkers within the organizational entity, while negative organizational treatments (i.e., low feedback quality) symbolize the employee’s minimal value to the organization (Restubog et al., 2008). Accordingly, when leader delivers teleworkers with high-feedback quality, teleworkers will feel appreciated and valued by the organization, which allows them to closely define themselves concerning what the organization represents (De Ruiter et al., 2018). As pointed out in De Ruiter et al. (2018), when an organization fulfills its obligations, it positively influences teleworkers’ psychological bond with the organization, and thus sense of belongingness from employees will be cultivated. Thus, we propose as:

*Hypothesis 2:* Perceived organizational support will mediate the positive relationship between feedback quality and teleworkers’ sense of belongingness.

### The Moderating Role of Conscientiousness

We realize that not all teleworkers like to use high-quality feedback to assess whether the organization is supportive of them. Conscientiousness is a component of the five-factor personality model (Costa and McCrae, 1988, 1992) and represents the degree to which individuals are dutiful, hard-working, persevering, and self-disciplined and tend to strive for achievement (Barrick et al., 2005; Resick et al., 2007). In the case of individuals assigned to the outside site (i.e., teleworkers), teleworkers with high conscientiousness will attach more importance to leaders’ feedback (VandeWalle, 2003; Vandewalle et al., 2019). We expect that conscientiousness will amplify the positive effects of feedback quality on perceived organizational support.

As suggested by organizational support theory, when the organization provides help and care for employees’ needs, employees will feel that the organization is supportive of them (Riggle et al., 2009). We propose that high conscientious teleworkers are more likely to recognize the value of work-related help (i.e., feedback) from the organization than low conscientious teleworkers due to their high expectations of work quality, and thus derive stronger organizational support from the feedback. Specifically, conscientious individuals value personal achievement more, so they care more about the high quality of work (Hohnemann et al., 2022), are more motivated (Huang et al., 2017), and are hard-working (Eissa, 2020). Thus, highly conscientious individuals are more concerned with achievement-related conditions, such as a sense of accomplishment (Tu et al., 2020), and are inclined to expend energy on conquering the work-related problems that they encounter (Eissa, 2020). That is, feedback becomes a more valuable asset that is needed to help them improve their work better for high conscientious employees. Conversely, teleworkers with less conscientiousness were not highly concerned about achievement at work, so the delivered low feedback quality may not cause a noticeable disturbance for them. Hence, we hypothesize as:

*Hypothesis 3:* Conscientiousness will moderate the direct effect of feedback quality on perceived organizational support. The effect will be stronger for teleworkers with high conscientiousness relative to low.

### Moderated-Mediation Model

To integrate these relationships, a moderated-mediation model is proposed. We propose that conscientiousness plays a moderating role in the indirect relationship between feedback quality and teleworkers’ sense of belongingness *via* perceived organizational support. As mentioned before, compared with low levels of conscientiousness, teleworkers with high levels of conscientiousness put more value on high-quality feedback. Thus, teleworkers with high levels of conscientiousness will perceive stronger organizational support from high-quality feedback, which further leads them to feel a stronger sense of acceptance and inclusion in the organization as a part of the organization. We hypothesize as:

*Hypothesis 4:* Conscientiousness will moderate the indirect effect of feedback quality on teleworkers’ sense of belongingness *via* perceived organizational support. The effect will be stronger when conscientiousness is high relative to low.

## Materials and Methods

### Samples and Procedures

The participants for this research were recruited from a Chinese information technology company and more than 3,000 employees, with customers all over the country in a wide range of industries. We chose this company’s teleworkers as our research subjects. Teleworking is defined as “work carried out in a location where remote from central offices or production facilities, the worker has no personal contact with co-workers there, but is able to communicate with them using new technology” (Di Martino and Wirth, 1990: 530). All the teleworkers had an area specifically devoted to their office space and the technological equipment necessary (i.e., PC) to carry out their job. However, the teleworkers had no set time to visit the office and face-to-face contact was minimal. They engaged in work away from the office location three or more days a week, which is defined as high-intensity telework in Belle et al. (2015).

This organization has around 2000 teleworkers. We distributed the electronic questionnaires by a random employee WeChat group of 500 teleworkers with the help of HR, which is a group formed by HR to facilitate daily management. We stated that the survey was a research study on the employees’ daily work and that the data would only be used for academic research, noting that it is anonymous and will not be personal. Moreover, we stated that the survey was divided into three waves at around two-week intervals. In the last question of each questionnaire, we asked the participants to fill in the last four digits of their phone numbers to match the responses and reassure the participants to provide more truthful answers (Podsakoff et al., 2003). We also emphasized that if they would like to know the results of the study in the future, they could leave an email in their questionnaires.

To ensure a two-week interval, we left the questionnaire system open for 3 days for each collection, which also allowed participants sufficient time to complete it. In the first-wave survey (T1), 422 employees completed the survey of feedback quality delivered by their leaders, conscientiousness, and personal information. In the second-wave survey (T2), the questionnaire of perceived organizational support was sent to the same WeChat group, and 380 participants completed it. In the third-wave survey(T3), 334 participants in the same WeChat group completed the questionnaire of sense of belongingness. We screened the questionnaires in the following ways. First, participants had to be teleworkers; second, the questionnaire was filled out completely; and third, the questionnaire was matched by the last four digits of their phone numbers. A 329 matched data were included in the last sample, with a 65.80% response rate. The average age was 29.89 years (*SD* = 6.58). A 200 participants were men. Education level for high school diploma or below, college diploma, bachelor’s degree, master’s degree, doctoral degree, and above were 3.3, 5.5, 46.8, 43.2, and 1.2%, respectively. Participants’ average dyadic tenure with leaders was 2.23 years (*SD* = 1.92). The average organizational tenure was 3.19 years (*SD* = 3.76).

### Measures

All survey items were translated into Chinese according to the back-translation procedure in Brislin’s (1986) study. A five-point Likert scale was used, ranging from 1 (=strongly disagree or to no extent) to 5 (=strongly agree or to a very large extent).

#### Feedback Quality

Feedback quality was measured using a five-item subscale (*α* = 0.90) from Steelman et al. (2004). Participants were asked to indicate the extent to which they agreed that a specific statement reflected the feedback practices from their direct leaders. One sample item was “the feedback I obtain from my direct leader is helpful.”

#### Conscientiousness

Employees rated their conscientiousness using five items (*α* = 0.89) from Singh (2019). One sample item was “I am always prepared for work.”

#### Perceived Organizational Support

To measure perceived organizational support, we used the eight items (*α* = 0.89) taken from Rhoades et al. (2001). One sample item was “help is available from my organization when I have a problem.”

#### Sense of Belongingness

Employees rated their sense of belongingness to the organization they had a contract with on the survey comprising five items (*α* = 0.88) from Hoogervorst et al. (2012). One sample item was “Please rate the extent to which you feel ‘valued’ by your company.”

#### Control Variables

Besides controlling for demographic variables (gender, age, and education level), we also controlled employee tenure in the organization and dyadic tenure (the leader–subordinate relationship) in the following analysis, which have been found to be essential for employees’ sense of belongingness by Ashforth et al. (2013).

## Results

### Descriptive Statistics and Confirmatory Factor Analysis

Means, standard deviations, and bivariate correlations are presented in Table 1. The preliminary analysis showed that feedback quality was positively related to perceived organizational support (*r* = 0.41, *p* < 0.01) and sense of belongingness (*r* = 0.50, *p* < 0.01), and perceived organizational support is positively related to sense of belongingness (*r* = 0.47, *p* < 0.01). Moreover, results showed that only age, education, and dyadic tenure are correlated to perceived organizational support or sense of belongingness. Thus, following the recommendation of Becker et al. (2016), we controlled the effects of age, education, and dyadic tenure in the following SEM analysis.

**Table 1:** Correlations and descriptive statistics.

	Mean	SD	1	2	3	4	5	6	7	8	9
1. Gender	1.39	0.49									
2. Age	29.89	6.58	−0.07								
3. Education	3.33	0.75	0.05	−0.14^**^							
4. Tenure	3.19	3.76	0.09	0.66^**^	−0.12^*^						
5. Dyadic tenure	2.23	1.92	0.14^*^	0.47^**^	−0.12^*^	0.76^**^					
6. Feedback quality	3.86	0.81	0.05	0.12^*^	−0.01	0.10	0.17^**^	(0.90)^a^			
7. Conscientiousness	2.36	0.72	−0.02	0.09	−0.13^*^	0.06	0.02	0.07	(0.89)		
8. Perceived organizational support	3.28	0.58	−0.08	0.12^*^	−0.14^**^	0.08	0.15^**^	0.41^**^	0.28^**^	(0.89)	
9. Sense of belongingness	3.76	0.66	0.02	0.09	−0.10	0.03	0.12^*^	0.50^**^	0.05	0.47^**^	(0.88)

N = 329; Gender was coded “1” for men and “2” for women. Education was coded “1” for “high school diploma or below,” “2” for “college diploma,” “3” for “bachelor’s degree,” “4” for “master’s degree,” and “5” for “doctoral degree.” ^a^Reliability coefficients are reported along the diagonal. ^*^p < 0.05, ^**^p < 0.01.

We used Mplus 7.4 to conduct several confirmatory factor analyses, and the results are shown in Table 2. We conducted item parcels for all variables recommended by Little et al. (2002), and 12 parcels were generated for four variables. The results demonstrate that the hypothesized four-factor measurement model has a better fit (*χ*^2^ = 70.44, *df* = 48, TLI = 0.99, CFI = 0.99, RMSEA = 0.04, SRMR = 0.02) than any of the other three-factor models.

**Table 2:** Confirmatory factor analysis for discriminant validity.

Model	*χ^2^*(*df*)	CFI	TLI	RMSEA [90% CI]	SRMR	Δ*χ^2^* (Δ*df*)^a^
Four-factor model (FB, POS, SOB and CON)	70.44(48)	0.99	0.99	0.04[0.02, 0.06]	0.02	-
Three-factor model (FB and POS were combined)	726.68(51)	0.76	0.69	0.20[0.19, 0.21]	0.13	656.24(3)^***^
Three-factor model (FB and SOB were combined)	485.94(51)	0.85	0.80	0.16[0.15, 0.17]	0.10	415.5(3)^***^
Three-factor model (FB and CON were combined)	750.13(51)	0.75	0.68	0.20[0.19, 0.22]	0.15	679.69(3)^***^
Three-factor model (POS and SOB were combined)	501.01(51)	0.84	0.80	0.16[0.15, 0.18]	0.11	430.57(3)^***^
Three-factor model (POS and CON were combined)	686.56(51)	0.78	0.71	0.20[0.18, 0.21]	0.13	616.12(3)^***^
Three-factor model (SOB and CON were combined)	734.93(51)	0.76	0.69	0.20[0.19, 0.22]	0.22	664.49(3)^***^

N = 329. ^a^The chi-square difference for each model reflects its deviation from the four-factor model. FB, Feedback quality; POS, perceived organizational support; SOB, sense of belongingness; CON, conscientiousness. ^*^p < 0.05, ^**^p < 0.01, and ^***^p < 0.001.

### Tests of Hypotheses

We used the SEM in Amos 26.0 with latent variables to test all hypotheses with bootstrapping procedure with 5,000 samples. To Hypotheses 1 and 2, we specified the direct and indirect effects of feedback quality on perceived organizational support and sense of belongingness. Three demographic variables (i.e., age, education level, and dyadic tenure) were used to predict perceived organizational support and sense of belongingness. We found that the model fit of the mediating effect was acceptable (*χ*^2^ = 462.56, df = 177, *χ*^2^/df = 2.61, CFI = 0.92, TLI = 0.91, RMSEA = 0.07). Table 3 summarizes standardized direct effects with lower and upper bound limits. We found feedback quality was significantly related to sense of belongingness [*b* = 0.27, 95% CIs (0.15,0.42), *p* < 0.001], supporting Hypothesis 1. In terms of considering perceived organizational support as a mediation mechanism linking feedback quality and sense of belongingness, we found a significant positive indirect effect of feedback quality on sense of belongingness *via* perceived organizational support, as indicated by the 95% confidence intervals [CIs; *b* = 0.09, 95% CIs (0.07, 0.21), *p* < 0.001], which excluded 0. Therefore, Hypothesis 2 was supported.

**Table 3:** Standardized direct, indirect, and interaction effects with lower and upper bound limits.

Bootstrap method	Bias-corrected percentile method		
Structural paths	*b*	CI	*p*
Feedback quality→Sense of belongingness	0.27	[0.15,0.42]	0.000
Perceived organizational support→Sense of belongingness	0.25	[0.14,0.37]	0.000
Feedback quality→Perceived organizational support	0.38	[0.28,0.49]	0.000
Feedback quality→Perceived organizational support→Sense of belongingness	0.09	[0.07,0.21]	0.000
Feedback quality × Conscientiousness→Perceived organizational support	0.35	[0.23,0.43]	0.002

N = 329. CI: confidence interval; b: unstandardized regression weight, b-values are computed through bootstrapping procedure with 5,000 bootstrap samples. ^*^p < 0.05, ^**^p < 0.01, and ^***^p < 0.001.

**Figure 2:**
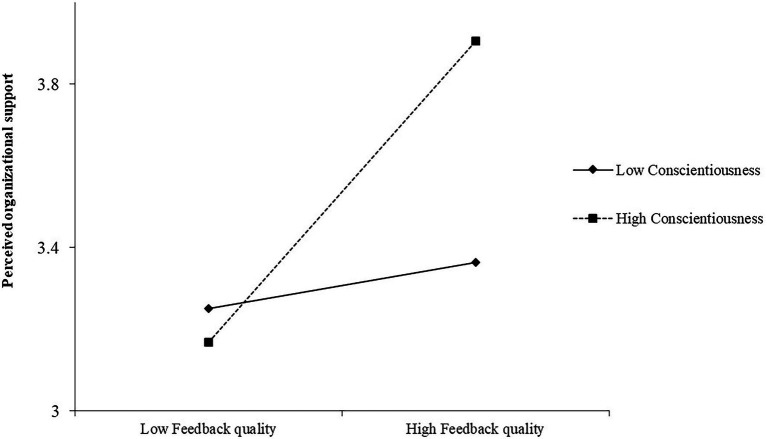
The direct effect of feedback quality on perceived organizational support when conscientiousness is low and high.

To test Hypothesis 3, we introduced conscientiousness as a moderator in the mediation model to predict perceived organizational support. All the predictors (i.e., feedback quality and conscientiousness) were mean-centered to reduce the potential for multicollinearity (Aiken et al., 1991). We found that the model fit of the moderated-mediation effect was also acceptable (*χ*^2^ = 840.31, df = 412, *χ*^2^/df = 2.04, CFI = 0.92, TLI = 0.91, RMSEA = 0.06). As shown in Table 3, the interaction term of feedback quality and conscientiousness was significantly related to perceived organizational support [*b* = 0.35, 95% CIs (0.23, 0.43), *p* < 0.001]. To assist with interpretation, the plot of the interaction effect is shown in Figure 2. Consistent with our expectation, simple slope analyses showed that feedback quality was more positively correlated with perceived organizational support when conscientiousness was at a high level (+1 *SD*; *b* = 0.46, *p* < 0.001) than when conscientiousness was at a low level (−1 *SD*; *b* = 0.07, *p* > 0.05), with a significant difference in the relationship magnitude (*difference* = 0.39, *p* < 0.001). Hypothesis 3 was thus supported.

Moreover, we examined the extent to which the overall mediation effect of perceived organizational support was conditionally influenced by the levels of conscientiousness. To test the difference of the conditional indirect effects under low and high levels of conscientiousness, Edwards and Lambert (2007) method, which has been widely used in later studies (Panaccio et al., 2014; Zheng et al., 2021), was followed. We used Model 7 in Hayes’ (2013) PROCESS macro with 5,000 bootstrap samples to test the moderated-mediation model. As expected, the indirect, positive effect of feedback quality on sense of belongingness *via* perceived organizational support was stronger when conscientiousness was at a high level [+1 *SD*; *effect size* = 0.16, Boot *SE* = 0.03, 95% CIs (0.10, 0.23)] than when conscientiousness was at a low level [−1 *SD*; *β* = 0.02, Boot *SE* = 0.01, 95% CIs (0.00, 0.05)], with a significant difference estimate [*difference* = 0.14, Boot *SE* = 0.02, 95% CIs (0.06, 0.14)]. Therefore, Hypothesis 4 was supported.

## Discussion

Research on sense of belongingness has gained widespread attention, but previous studies have emphasized the importance of co-workers (Thau et al., 2007) and organizational inclusive atmosphere (Shore et al., 2018) in improving office employees’ sense of belongingness. But empirical studies targeting teleworkers are uncommon. Especially, less attention has been paid to how feedback quality influences teleworkers’ sense of belongingness. In the current study, we drew on organizational support theory (Shanock and Eisenberger, 2006) to build and examine a theoretical model that explains why and when feedback quality may evoke teleworkers’ sense of belongingness—an experience where individuals feel themselves to be an integral part organization in the workplace. We found that feedback quality can foster employees’ sense of belongingness *via* perceived organizational support. A possible explanation of this finding is that feedback as a work-related resource, providing employees with quality feedback is a symbol of approval for employees and allows them to feel valued and nurtured by the organization which further makes them more willing to stay with the organization and belong to it. This finding also corroborates Riggle et al.’s (2009) view that in a supportive work environment, employees feel more included in the organization. Moreover, the results also indicated that perceived organizational support mediated partially the relationship between feedback quality and sense of belongingness. This is probably because organizational support theory is not the only way to link feedback quality to sense of belongingness, but it also re-emphasizes the importance of high-quality feedback.

Furthermore, we found teleworkers with high levels of conscientiousness than low levels were more likely to perceive organizational support and experience a sense of belongingness. Specifically, for teleworkers with high levels of conscientiousness relative to low levels, the positive direct relationship between feedback quality and perceived organizational support and the positive indirect relationship between feedback quality and sense of belongingness *via* perceived organizational support both became stronger. The finding strengthens the crucial value of teleworkers’ personality in shaping individuals’ prioritization of needs. It is clear in this study that the need for high-quality feedback is higher for teleworkers who are more conscientious.

### Theoretical Implications

This study extends the sense of belongingness literature in virtual employee management in many ways. First, based on the most fundamental needs of teleworkers, this study identifies feedback quality as the antecedents of influencing teleworkers’ sense of belongingness and verifies the important value of perceived organizational support in motivating teleworkers’ sense of belongingness. This study responds to the appeal and improves the attention to the value of feedback quality (Ashford et al., 2016), emphasizing that feedback has become a core resource for employee learning and self-developing, which further ensures employees’ eagerness to belong to the organization (Anseel et al., 2007).

Second, we found the backing for the mediating role of perceived organizational support between feedback quality and sense of belongingness. This study contributes to intrinsic mechanism exploration linking feedback quality to sense of belongingness. That is, for teleworkers, perceived organizational support is essential in helping them perceive their own as a part of the organization. This is in agreement with the findings of Wiesenfeld et al. (2001) and Shore et al. (2011), such perceived personal experience of getting support at work is a powerful force in bolstering their perception of belongingness to the organization. Moreover, scholars have investigated many employee outcomes that were influenced by organizational support (Ahmed et al., 2015), such as organizational commitment, employee engagement, job satisfaction, and turnover intentions. However, this study found a more specific result examining the impact of perceived organizational support on sense of belongingness.

Finally, we introduce teleworkers’ conscientiousness as a crucial boundary factor to analyze the influence of feedback quality. It confirmed that conscientious teleworkers pay more attention to feedback quality for effectively solving the problems faced at work and provides a more detailed process of how feedback quality influences teleworkers’ outcomes, which expands the research related to conscientiousness in the field of feedback.

### Practical Implications

The present research’s overall outcomes have several practical implications for organizations. First, this study clearly emphasized the importance of giving teleworkers high-quality feedback, because receiving high-quality feedback can trigger positive attitudes, cognitions, and behaviors (Steelman et al., 2004; Wang et al., 2015) such as perceived organizational support and sense of belongingness. For teleworkers, physical distance causes information deficits (Handke et al., 2021), social and professional isolation (Nicklin et al., 2016; Wang et al., 2020). They experience a great deal of ambiguity. Leaders become the merely important bridge between teleworkers and the organization. A leader of teleworkers should give tailored and thoughtful feedback (Jarvenpaa et al., 1998; Bell and Kozlowski, 2002). Thus, daily feedback from the leader for teleworkers becomes crucial information for employees to reduce job uncertainty, learn and improve job performance (Virick et al., 2010). For teleworkers, it is recommended that leadership and management training programs focus on learning their feedback needs (Marstand et al., 2018). Furthermore, as mentioned by Riggs and Porter (2017), leaders should strive to discuss their behaviors with teleworkers (e.g., giving high-quality feedback) to better meet teleworkers’ needs.

Second, our findings also highlight the important role of perceived organizational support for teleworkers in transmitting the influence of feedback quality on sense of belongingness. On the one hand, effective management communication (i.e., giving high-quality feedback) is essential to signal that the organization cares about the wellbeing and values the contributions of its employees (Allen, 1992; Neves and Eisenberger, 2012). At the same time, managers can empathize with their teleworkers and understand their concerns and difficulties. In addition, research has shown that a variety of organizational practices bring employees and organizations closer together and benefit employees’ perception of organizational support. For example, DeConinck (2010) found that organizational justice has a positive relationship with perceived organizational support through employees’ perception that the organization cares about their welfare. Human resource practices (i.e., high-performance work practices) were also proved to enhance perceived organizational support by investing in the skills and abilities of employees, designing work in a way that facilitates employee collaboration in problem solving, and providing incentives to enhance motivation (Gavino et al., 2012). The power and influence of the broad organization relative to factors of individual levels may create more advantages in fostering employees’ perception of organizational support, which in turn promotes their sense of belongingness.

Finally, the research results show that teleworkers with high levels of conscientiousness are more likely to perceive organizational support and a sense of belongingness. It indicates that conscientiousness should become an evaluation criterion in the process of recruiting and selecting teleworkers in the organization (Eissa, 2020; Tu et al., 2020), given that this personality for teleworkers is imperative to cherish more job-related resources from organizations, such as job feedback.

### Limitations and Future Research

This study has several limitations worth discussing. First, the self-report method was used to measure all variables; thus, the common method deviation is worrisome. We collected data from three different times to a certain extent and alleviated the concern of common method bias. However, future research could solve common method deviation problems by using some objective data such as leveraging the turnover rate of teleworkers to detect their sense of belongingness.

Second, along with perceived organizational support theory, we found perceived organizational support as the mechanism connecting feedback quality to teleworkers’ sense of belongingness. However, the analysis results showed that perceived organizational support did not play a fully mediated role; thus, other potential mechanisms cannot be excluded. For example, the level of feedback quality may serve as a kind of motivation/stressor that triggers teleworkers’ excitement/anxiety, positively correlated to positive/negative affectivity based on affective event theory.

Finally, future research should consider other buffers. For example, high-quality leader-member exchange characterizes individuals as trustworthy, respectful, loyal, and having a mutual obligation with their leaders to arouse their positive affectivity, which can strengthen the positive effect of satisfying experiences (i.e., high-quality feedback) and reduce the negative effect of unpleasant experiences (Kreemers et al., 2018; Qian et al., 2020).

## Conclusion

The present study used a sample of teleworkers to explore the underlying relationship linking feedback quality to sense of belongingness. Since most teleworkers work independently outside the organization, their need for a sense of belongingness is particularly pronounced. The hypothesized moderated-mediation model demonstrated that teleworkers who received high-quality feedback delivered by their direct leaders would perceive support from the organization and subsequently felt a sense of belongingness. Especially, high conscientious teleworkers relative to low could perceive stronger organizational support from the delivered feedback from leaders, which in turn experienced a stronger sense of belongingness. The findings emphasize the importance of high-quality feedback and perceived organizational support in facilitating teleworkers’ sense of belongingness, and those relationships are even more obvious for high conscientious individuals rather than low. Finally, these findings are valuable in helping HR practitioners and supervisors to create a job-related environment that effectively builds teleworkers’ sense of belongingness.

## Data Availability Statement

The raw data supporting the conclusions of this article will be made available by the authors, without undue reservation.

## Author Contributions

YL, NX, QY, and ZL: research design. NX and QY: data collection. YL and ZL: data analysis. YL: writing of the original draft. YL, NX, QY, ZL, and ZT revising the article. All authors contributed to the article and approved the submitted version.

## Funding

The authors disclosed receipt of the following financial support for the research, authorship, and/or publication of this article: This study was funded by the National Natural Science Foundation of China (grant number 72072096), Scientific Research and Innovation Projects for Tianjin Postgraduates (grant numbers 2019YJSB087 and 2020YJSB013), the China Scholarship Council Project (grant number 202006200088), and Philosophy and Social Science Foundation of Heilongjiang Province (20GLC206).

## Conflict of Interest

The authors declare that the research was conducted in the absence of any commercial or financial relationships that could be construed as a potential conflict of interest.

## Publisher’s Note

All claims expressed in this article are solely those of the authors and do not necessarily represent those of their affiliated organizations, or those of the publisher, the editors and the reviewers. Any product that may be evaluated in this article, or claim that may be made by its manufacturer, is not guaranteed or endorsed by the publisher.
